# Mesenchymal stem cell-derived exosomes promote hepatic regeneration in drug-induced liver injury models

**DOI:** 10.1186/scrt465

**Published:** 2014-06-10

**Authors:** Cheau Yih Tan, Ruenn Chai Lai, Winnie Wong, Yock Young Dan, Sai-Kiang Lim, Han Kiat Ho

**Affiliations:** 1 Department of Pharmacy, National University of Singapore, Block S4 18 Science Drive 4, Singapore 117543, Singapore; 2 Department of Gastroenterology, National University Hospital, 5 Lower Kent Ridge Road, Singapore 119074, Singapore; 3 Institute of Medical Biology, Agency for Science Technology and Research, Singapore 138648, Singapore

## Abstract

**Introduction:**

Mesenchymal stem cell-conditioned medium (MSC-CM) has been shown to have protective effects against various cellular-injury models. This mechanism of protection, however, has yet to be elucidated. Recently, exosomes were identified as the active component in MSC-CM. The aim of this study is to investigate the effect of MSC-derived exosomes in an established carbon tetrachloride (CCl_4_)-induced liver injury mouse model. This potential effect is then validated by using *in vitro* xenobiotic-induced liver-injury assays: (1) acetaminophen (APAP)- and (2) hydrogen peroxide (H_2_O_2_)-induced liver injury.

**Methods:**

The exosomes were introduced concurrent with CCl_4_ into a mouse model through different routes of administration. Biochemical analysis was performed based on the blood and liver tissues. Subsequently the exosomes were treated in APAP and H_2_O_2_-toxicants with *in vitro* models. Cell viability was measured, and biomarkers indicative of regenerative and oxidative biochemical responses were determined to probe the mechanism of any hepatoprotective activity observed.

**Results:**

In contrast to mice treated with phosphate-buffered saline, CCl_4_ injury in mice was attenuated by concurrent-treatment exosomes, and characterized by an increase in hepatocyte proliferation, as demonstrated with proliferating cell nuclear antigen (PCNA) elevation. Significantly higher cell viability was demonstrated in the exosomes-treated group compared with the non-exosome-treated group in both injury models. The higher survival rate was associated with upregulation of the priming-phase genes during liver regeneration, which subsequently led to higher expression of proliferation proteins (PCNA and cyclin D1) in the exosomes-treated group. Exosomes also inhibited the APAP- and H_2_O_2_-induced hepatocytes apoptosis through upregulation of Bcl-_xL_ protein expression. However, exosomes do not mitigate hepatocyte injury via modulation of oxidative stress.

**Conclusions:**

In summary, these results suggest that MSC-derived exosomes can elicit hepatoprotective effects against toxicants-induced injury, mainly through activation of proliferative and regenerative responses.

## Introduction

Liver is one of the few organs in the body that possesses an immense capacity to regenerate through the replication of mature functioning liver cells [1]. This protective response is of clinical significance particularly during liver injury. However, when the injury progresses into a state of functional impairment, it can overwhelm or even inhibit the intrinsic regenerative potential, leading to severe consequences that include acute liver failure or death [2]. Today, xenobiotic (drugs)-induced liver injury accounts for more than 50% of acute liver failure in the United States and has become a major clinical problem [3]. To overcome this issue, we must explore potential therapeutic agents that can prevent further damage to the injured liver cells. Alternatively, agents that can stimulate remnant liver cells to regenerate could also help to restore liver function in a timely manner and to avert longer-term sequelae.

Mesenchymal stem cell (MSC)-based therapy has been extensively investigated in the area of regenerative medicine for different organs (heart, kidney, lung, and liver) because of its ability to differentiate and transdifferentiate into various tissue types (for example, from osteoblasts into adipocytes and chondrocytes) [4], stimulate regeneration, and repair damaged tissue/organs [5]. However, recent reports showed that the bioactive soluble factors found in MSC-conditioned medium (MSC-CM) can stimulate host responses for repair and also reduce the liver-injury level in several different injury models, even without MSC [6-8]. As compared to cell-based therapies, MSC-CM (non-cell-based) therapies are generally preferred because they are less likely to trigger immune response, rendering them safer to use. In addition, they are more amenable to reformulation to support different routes of administration.

Recently, proteomic and computational analysis on the MSC-CM revealed that the secretome in this condition medium (CM) could exert potential modulating effects on tissue repair and regeneration in heart, kidney, and liver [9]. More important, it was demonstrated that the observed cardiac-function preservation was derived from exosomes, a fraction of particle size with 55–100 nm, bi-lipid membrane secreted microvesicle purified from the MSC-CM [10]. However, it remains to be determined if these size-purified exosomes are able to enrich the hepatoregenerative effect found previously in the conditioned medium, and whether they are efficacious in overcoming liver injury. Furthermore, their ability to respond to different mechanisms of liver injury has not been investigated.

Therefore, this work set out first to explore the potential therapeutic effect in an established CCl_4_-induced acute liver-injury mouse model followed by characterizing this potential effect by using liver cell-culture models of well-defined xenobiotic-induced liver injury.

## Materials and methods

### Materials and reagents

Dexamethasone, nicotinamide, gentamicin, HEPES, NaCl, EDTA, glycerine, Triton-X, sodium fluoride, sodium orthovanadate, phenylmethanesulfonyl fluoride (PMSF), aprotinin, 3-(4,5-dimethylthiazol-2-yl)-2,5-diphenyltetrazolium bromide (MTT), acetaminophen (APAP), hydrogen peroxide (H_2_O_2_), carbon tetrachloride (CCl_4_), and olive oil were obtained from Sigma Chemical (St. Louis, MO, USA). Dulbecco modified Eagle medium/Ham F12 (DMEM/F12), and Superscript III First-strand Synthesis System are products of Invitrogen (Carlsbad, CA, USA). Insulin, transferrin, and selenium (ITS) is from BD (Franklin Lakes, NJ, USA). Dimethylsulfoxide (DMSO) was obtained from Merck (Darmstadt, Germany). SYBR Green PCR master mix was obtained from Applied Biosystems (Warrington, UK). RNeasy mini kit is a product of Qiagen (Hilden, Germany). Phosphate-buffered saline (PBS) and all primers were synthesized by 1st BASE Oligos (Singapore). Primary antibodies were purchased from the following companies: phospho (Tyr705)-STAT 3, proliferating cell nuclear antigen (PCNA), NF-κB p65, cyclin D and cyclin E, Cell Signaling Technology (Danvers, MA, USA); hepatocyte growth factor (HGF), Abcam (Cambridge, UK). MSC-derived exosomes was prepared and purified as described previously [10].

### Animals and diets

Six-week-old male C57BL/6 mice (21 to 27 g) were supplied by the Lab Animals Centre, Singapore. Animals were housed in animal-holding units in National University of Singapore (NUS) at a constant temperature in a 12/12-hour light/dark cycle. All animal procedures were carried out according to a protocol approved by the National University of Singapore Institutional Animal Care & Use Committee, Protocol number 123/09. The mice were acclimatized for 2 weeks before use; the mice were 8 weeks of age at the time of experiments. Before the experiment, mice were allowed free access to food and water.

### Preparation of MSC-derived exosomes

The collection of fetal tissue was carried out under a KK Women’s and Children’s Hospital (KKH) IRB-approved protocol (EC200804062) [11]. Only patients who had already consented to Termination of Pregnancy (TOP) in theKKH Outpatient Clinic were recruited. Recruitment was carried out in strict adherence to KKH IRB regulations to ensure the patient’s rights and privacy, and to provide confidential counseling for the patient’s fully informed consent to voluntary donation. The patients gave their informed consent for fetal tissue to be collected and used in this study. The hESC-derived HuES9.E1 MSCs were used for the production of MSC secretion in the form of CM, as described [10].

To harvest MSC secretion, 80% of the confluent HuES9. E1 cultures were washed with PBS, transferred to a chemically defined, serum-free culture medium for an overnight incubation, washed with PBS, and cultured in fresh, chemically defined, serum-free culture medium for 3 days. The CM was collected, clarified by centrifugation, and was concentrated 50× by tangential flow filtration (TFF) by using a membrane with a 100-kDa MWCO (Sartorius, Goettingen, Germany). CM was fractionated by high-performance liquid chromatography (HPLC) (TSK Guard column SWXL, 6 × 40 mm and TSK gel G4000 SWXL, 7.8 × 300 mm, Tosoh Corp., Tokyo, Japan).

Exosomes were collected from the first peak of the elution and concentrated by using 100 kDa MWCO filter (Sartorius). The identity of the exosomes used in this study had been previously characterized by Lai *et al.*[10]. Exosomes were filtered with a 0.22-μm filter and stored in a -20°C freezer until use.

### CCl_4_ and exosomes administration

All mice received a single dose of CCl_4_ (3% vol/vol in olive oil) at 0.05 ml/kg body weight by intraperitoneal injection (i.p*.*). 0.4 μg (100 μl) exosomes or 100 μl PBS were administered through intrasplenic injection (i.s.) for the treatment group and the vehicle-control group, respectively. All mice were killed 24 hours after CCl_4_, blood, and livers were collected. The aspartate aminotransferase (AST) and alanine aminotransferase (ALT) activities in the serum were analyzed by using Cobas 4000 analyzer series (Roche, Basel, Switzerland).

### Cell lines and culture conditions

Three different types of hepatocytes were used in the study of cell viability. TAMH, an immortalized mouse hepatocyte line derived from transgenic MT42 male mice overexpressing TGF-α [12], was used as a metabolically competent liver cell line that reproduced features of cytotoxicity to support this investigation, whereas THLE-2 is an immortalized primary human hepatocyte that expresses phenotypic characteristics of normal adult liver epithelial cells [13].

In contrast with the normal cell line, we also investigated the effect of exosomes in HuH-7, a well-differentiated human hepatocarcinoma cell line. This comparison will help to strengthen the evidence of therapeutic effect of exosomes against different liver-injury model in various liver cell types.

Immortalized murine transforming growth factor alpha (TGF-α) transgenic hepatocyte (TAMH) cells [14] (obtained as a kind gift from Prof Nelson Fausto, University of Washington, Seattle, WA, USA), were maintained in DMEM/F12 supplemented with 5 mg/ml insulin, 5 mg/ml transferrin, 5 ng/ml selenium, 100 n*M* dexamethasone, 10 m*M* nicotinamide, and 0.01% (vol/vol) gentamicin. HuH-7 obtained from HSRRB (Tokyo, Japan), a human hepatocarcinoma cell line was grown in Dulbecco modified Eagle medium (DMEM) (Sigma) supplemented with 10% fetal bovine serum (FBS) (Hyclone). THLE-2 obtained from ATCC (Manassas, VA, USA), a human normal liver cell, was cultured in LHC-9 medium supplemented with 10% FBS (Hyclone). The flasks used were pre-coated with a mixture of 0.01 mg/ml fibronectin, 0.03 mg/ml bovine collagen type I, and 0.1 mg/ml bovine serum albumin (BSA) dissolved in LHC-9 basal medium.

All cells were maintained at 37°C in humidified 95% air and 5% CO_2_ atmosphere, passaged and seeded at 80% to 90% confluency.

### *In vitro* cell-viability assay

With 96-well plates, TAMH cells were seeded at a density of 6 × 10^3^ cells/well in 200 μl DMEM/F12 medium, whereas THLE-2 cells were seeded at a density of 1 × 10^4^ cells/well in 200 μl LHC-9 medium overnight. HuH-7 cells were seeded at a density of 5 × 10^3^ cells/well for 72-hour treatment, and 7.5 × 10^3^ cells/well for 24-hour treatment in 200 μl DMEM medium overnight. TAMH, HuH-7, and THLE-2 cells were treated with various concentrations of exosomes in their respective culturing medium for 24 or 72 hours. Cell-viability assays were performed after the respective incubation time to determine the cytotoxicity of exosomes. In TAMH cells, 2 m*M* APAP or 350 μ*M* H_2_O_2_ was treated in the TAMH cells concurrent with 0.05 μg/ml or 0.1 μg/ml of exosomes for 24 hours before performing the cell-viability test. HuH-7 and THLE-2 cells were treated with 5 m*M* and 3.5 m*M* APAP, respectively, with 0.05 μg/ml or 0.1 μg/ml of exosomes for 72 hours before performing the cell-viability test.

PBS was used as background for exosomes treated in the control groups. After incubation of toxicants with different concentrations of exosomes, the cell viability was evaluated with MTT assay. Then 20 ml of 5 mg/ml MTT was dissolved in PBS. After the incubation period, the media was aspirated, and the formazan crystals in cells were dissolved in 200 μl of DMSO and 25 μl of Sorenson buffer [15]. The absorbance was read at 570 nm by using a Tecan microtiter plate reader. Cell-viability percentage was expressed as a ratio of cells exposed to different concentrations of toxicants with those of vehicle controls.

### Reverse transcription and quantitative real-time polymerase chain reaction

In a six-well plate, TAMH cells were seeded at a density of 0.5 × 10^6^ cells/well and treated with 2 m*M* APAP or 350 μ*M* H_2_O_2_ concurrent with 0.05 mg/ml or 0.1 mg/ml of exosomes for 24 hours. All cells were harvested for total cell RNA extraction by using RNeasy columns (Qiagen, Valencia, CA, USA). The quality and quantity of total RNA was determined with NanoDrop (Thermo, Wilmington, DE, USA), ensuring that RNAs with OD 260/280 > 1.80 were used. First-strand cDNA was synthesized from 1 μg total RNA by using Superscript First-Strand Synthesis System, according to the protocols of the manufacturer. qRT-PCR was performed by using BioRad CFX96 real-time PCR system with SYBR Green master mix and primers, as shown in Table 1. The thermal-cycling condition comprised an initial denaturation at 95°C (10 minutes), followed by 40 cycles at 95°C (15 seconds) and 60°C (60 seconds). Melting curves were generated at the end of 40 cycles to verify the purity of the PCR product. Data were obtained as average Ct values, and normalized against the geometric mean of GAPDH endogenous controls as Ct. Transcript differences between the exosomes-treated group and the nontreated group were measured as fold changes by using the comparative Ct method. Statistics were performed on Ct by using REST software (Qiagen).

**Table 1:** Sequences of primers used in real-time PCR reaction

**Gene**	**Forward primer (5ʹ- > 3ʹ)**	**Reverse primer (5ʹ- > 3ʹ)**
*In vitro* hepatocytes (TAMH cells)/*In vivo* liver (mouse)		
*TNF-α*	ATG AGC ACA GAA AGC ATG ATC	TAC AGG CTT GTA ACT CGA ATT
*IL-6*	AGT TGC CTT CTT GGG ACT GA	TCC ACG ATT TCC CAG AGA AC
*iNOS*	CAC CTT GGA GTT CAC CCA GT	ACC ACT CGT ACT TGG GAT GC
*COX-2*	CTC CCT GAA GCC GTA CAC AT	GCT CGG CTT CCAA GTA TTG AG
*MIP-2*	AAG TTT GCC TTG ACC CTG AA	AGG CAC ATC AGG TAC GAT CC
*HO-1*	CAG GTG ATG CTG ACA GAG GA	ATG GCA TAA ATT CCC ACT GC
*Gpx4*	CCG GCT ACA ACG TCA AGT TT	CGG CAG GTC CTT CTC TAT CA
*Gsr*	ACC ACG AGG AAG ACG AAA TG	GGT GAC CAG CTC CTC TGA AG
*MnSOD*	GGC CAA GGG AGA TGT TAC AA	CCT TGG ACT CCC ACA GAC AT
*GAPDH*	GGC ATT GCT CTC AAT GGAC AA	CCG AGGT TGG GAT AGG GCC

### Western blots

Mouse liver tissue was homogenized by using glass dounce homogenizer. Proteins were lysated with 800 μl of lysis buffer containing 50 m*M* HEPES pH 7.5, 150 m*M* NaCl, 1 m*M* EDTA, 10% glycerine, 1% triton-X, 0.5 *M* sodium fluoride, 100 m*M* sodium orthovanadate, 100 m*M* PMSF, and 20 mg/ml aprotinin. TAMH cells were seeded at 2 × 10^6^ in a 10-cm dish with DMEM/F12 medium overnight. The medium was changed to DMEM/F12 low glucose and cultured for 2 days. Cells were then treated with respective concentrations of APAP or H_2_O_2_ and exosomes for 24 hours. All cells were harvested for Western blot analysis. Cell pellets were lysed with 100 μl of RIPA lysis buffer containing 50 m*M* Tris, 150 m*M* NaCl, 0.1% SDS, 0.5% sodium deoxycholate, 1% NP-40, 0.5 *M* sodium fluoride, 100 m*M* sodium orthovanadate, 100 m*M* PMSF, and 20 mg/ml aprotinin. The 40 mg total protein per mouse sample and 10 μg per cell sample were resolved in 10% SDS-PAGE and immobilized on PVDF membrane. The membranes were blocked in 5% milk for 1 hour and incubated in respective primary antibodies overnight (pSTAT3, 1:1,000; NF-κB, 1:2,000; Bcl-_xL_ 1:1,000; PCNA, 1:10,000; and cyclin D1, 1:1,000), followed by respective secondary antibody (1:10,000) incubation for 1 hour. All membranes were visualized by using chemiluminescence substrate (Pierce Biotechnology, Rockford, IL, USA). The band intensities were normalized against actin and were quantified by using ImageJ software.

### Statistical analysis

Data were expressed as means ± standard error of means (SEM) and analyzed by using Student two-tailed *t* test. Statistical significance of the difference was accepted at *P*-values of less than 0.05.

## Results

### Exosomes reduced liver-injury level in ALT and AST after CCl_4_ treatment *in vivo*

**Figure 1:**
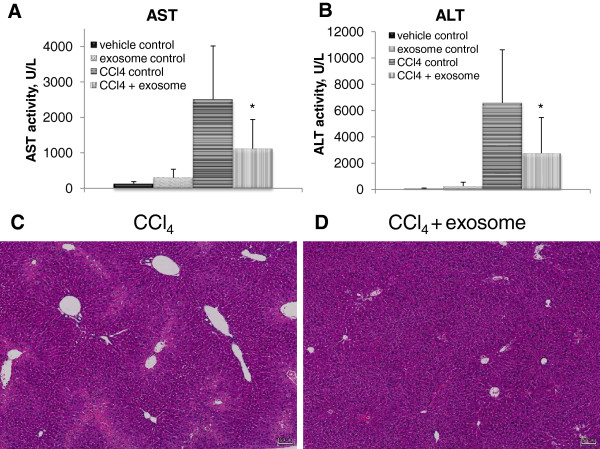
**Effect of exosomes on biochemical parameters and hepatocyte injury after CCl**_**4 **_**treatment *in vivo*.** Serum **(A)** aspartate aminotransferase (AST) and **(B)** alanine aminotransferase (ALT) levels were measured after 24-hour dosing of CCl_4_ with or without exosomes administration (*n* = 6 per group; *P < 0.05 versus of CCl_4_ control for both AST and ALT). Standard hematoxylin and eosin (H&E) staining for liver tissue harvested after 24 hours of **(C)** CCl_4_ administration and **(D)** CCl_4_ with exosomes treatment. Obvious hepatic necrosis appeared in the section from a CCl_4_-treated animal. Original magnification × 80; scale bar, 100 μm.

A preliminary experiment revealed that serum AST and ALT gradually increased and peaked at 24 hours after a single dose of CCl_4_ (data not shown). Thus, AST and ALT were measured at 24 hours after respective treatments. Mice receiving 0.05 ml/kg CCl_4_ demonstrated significant increases in AST and ALT within 24 hours of dosing. In mice treated with CCl_4_ and exosomes, the increase of AST and ALT after 24 hours was significantly less than the PBS-treatment group (Figure 1A). Histologically, the hematoxylin and eosin (H&E) staining of liver tissues for the CCl_4_ group showed moderate hepatic necrosis, whereas minimal necrotic cells were observed in the exosomes treatment group (Figure 1B).

### Exosomes induced hepatocyte-regenerative genes expression in liver tissue after CCl_4_-induced injury

**Figure 2:**
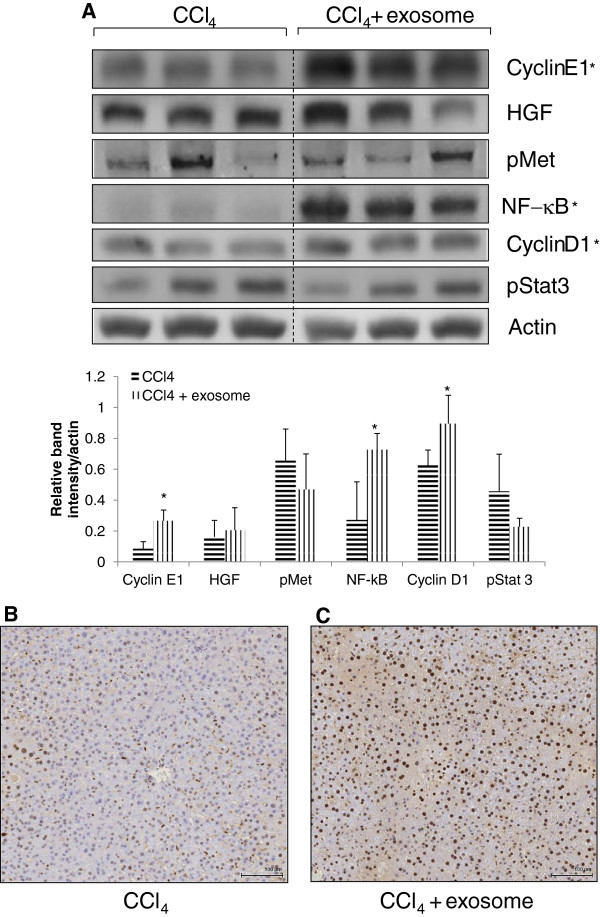
**Effect of exosomes on hepatocyte proliferation after CCl**_**4**_**-induced injury in mice. (A)** Expression of cyclin E, HGF, pMet, NF-κB p65, cyclin D1, and phosphorylated STAT3 was determined by immunoblotting after 24 hours of CCl_4_ administration with or without exosomes (*n* = 6 per group (three per group were shown); *P < 0.05 versus CCl_4_ control). The relative densities of all the proteins bands were normalized to actin in the same samples. Livers were processed for immunohistochemistry on PCNA to quantify hepatocyte proliferation after 24 hours of **(B)** CCl_4_ administration and **(C)** CCl_4_ with exosomes treatment. Original magnification × 200; scale bar, 100 μm.

To investigate the regenerative effect of exosomes, few of the transcription factors and growth factors expression involved in different stages of the cell cycle were examined by using immunoblot assays on the liver tissue. As can be seen from Figure 2A, the exosomes treatment group showed significant upregulation of the hepatic gene expressions of NF-κB, cyclin D1, and cyclin E in contrast with the untreated. No significant upregulation was observed for HGF and p-STAT3 gene expression. The activation of proliferation was also demonstrated by the higher number of PCNA^+^-stained cells in the exosomes treatment group (Figure 2C) as compared with the PBS-treatment group (Figure 2B).

### Exosomes exerted no intrinsic cytotoxicity while demonstrating increased cell viability in TAMH, THLE-2, and Huh-7 hepatocytes after APAP- or H_2_O_2_-induced injuries

**Figure 3:**
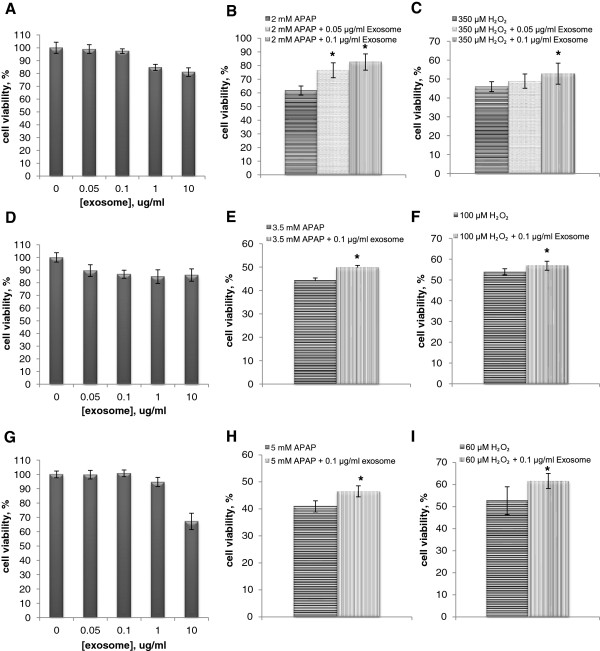
**Effect of exosomes in cell viability after APAP- and H**_**2**_**O**_**2**_**-induced injury.** Experiments were performed in TAMH **(A-C)**, THLE-2 **(D-F)**, and Huh-7 **(G-I)** hepatocytes. **(A, D, G)** Cytotoxicity of exosomes at different concentrations in respective cell lines. 0.05 μg/ml and 0.1 μg/ml of exosomes were added to respective concentration of APAP **(B, E, H)** and H_2_O_2_**(C, F, I)**, and MTT were performed 24 or 72 hours later. Cell viability was normalized against vehicle control group and expressed in percentage (*n* = 6 per group; *P < 0.05 versus APAP or H_2_O_2_ non-exosomes-treatment group.

*In vitro* models were subsequently designed to validate the *in vivo* findings of exosomal protection and to gain further insights into its response toward different mechanisms of toxicity; APAP represents the liver-injury model caused by both covalent modification of protein targets and oxidative stress-mediated injury pathways [16], whereas H_2_O_2_ represents only the oxidative stress-induced liver-injury pathway. Three representative liver cell lines, TAMH, THLE-2, and HuH-7 cells, were subjected to these xenobiotic-induced injuries. The exosomes concentration used in this study was 0.1 μg/ml. This concentration was chosen as it had demonstrated cardioprotection in a previous study [10]. Further in our current evaluation, this concentration did not display any cytotoxicity across the three cell lines (Figure 3A,D,G). Cytoprotection against APAP- and H_2_O_2_-induced liver injury in TAMH cells was clearly demonstrated (Figure 3B,C). The same concentration of exosomes seemed to demonstrate better protection against APAP injury as compared with the H_2_O_2_-injury model. When cross-referenced and confirmed with THLE-2 and HuH-7, comparable results were observed. Treatment of 0.1 μg/ml exosomes demonstrated significantly higher cell viability in both toxicant-induced liver-injury models by using THLE-2 (Figure 3D-F) and HuH-7 when compared with untreated cells (Figure 3G-I).

### Exosomes regulated hepatocytes proliferation through inducing quiescent hepatocytes (G_0_) to re-enter the cell cycle (G_1_) at the priming phase after APAP- or H_2_O_2_-induced injury

**Figure 4:**
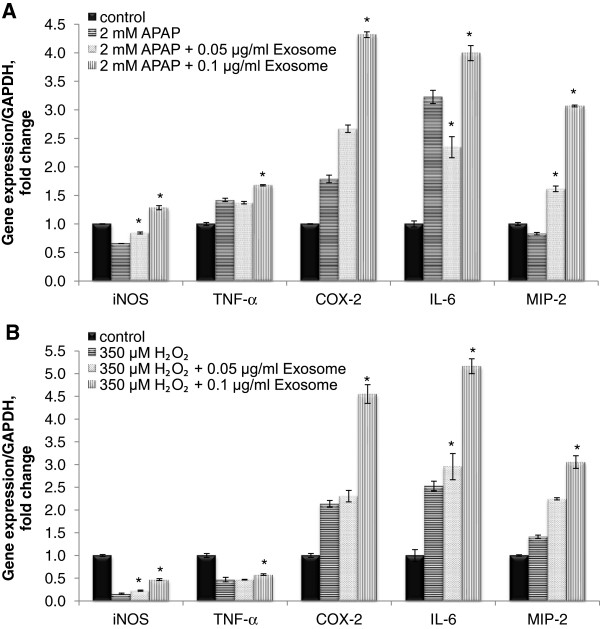
**Effect of exosomes in hepatoregenerative-expression genes on APAP- and H**_**2**_**O**_**2**_**-induced injury in hepatocytes.** Expression of regenerative-response genes after 24 hours of exosomes in **(A)** APAP and **(B)** H_2_O_2_ treatment was determined by quantitative real-time PCR and normalized against GAPDH expression of the same sample and presented as fold-increase over the controls. Bars represent means ± SEM. *P < 0.05 versus APAP or H_2_O_2_ nonexosomes treatment group.

By determining the modulation of early priming cytokines expression, an exosomal mechanism of cytoprotection could be elucidated. Hence, cell growth was first arrested by culturing in low-glucose DMEM/F12 medium for 2 days. Then, treatment with APAP or H_2_O_2_ was introduced, either with or without MSC exosomes. Finally, gene-expression levels of the priming factors were measured after 24 hours of these treatments. As shown in Figure 4A and B, 0.1 μg/ml of exosomes-treated group was observed to have elevated mRNA expressions of tumor necrosis factor alpha (TNF-α), interleukin 6 (IL-6), inducible nitric oxide synthase (iNOS), cyclooxygenase-2 (COX-2), and macrophage inflammatory protein 2 (MIP-2), as compared with the untreated group.

In the APAP-injury model, MIP-2 demonstrated the highest elevation with 3.7-fold, followed by 2.4-fold in COX-2 and twofold in iNOS. The elevation trend differed slightly in the H_2_O_2_-injury model, whereby the same amount of exosomes was found to increase iNOS expression the most significantly, by approximately 2.9-fold, followed by twofold increase in all three *MIP-2, COX-2*, and *IL-6* genes compared with the non-exosomes-treated group (Figure 4B).

### Exosomes induced transcription factors expression during the G_1_ phase of cell cycle after APAP- or H_2_O_2_-induced injury

**Figure 5:**
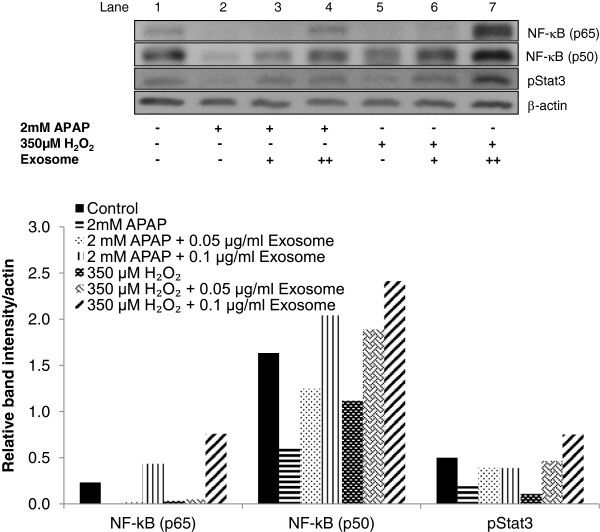
**Effect of exosomes in G**_**1**_**phase of the cell cycle after APAP- or H**_**2**_**O**_**2**_**-induced injury in hepatocytes.** Expression of NF-κB (p65 and p50) and phosphorylated STAT3 was determined by immunoblotting after 24-hour treatment in APAP- or H_2_O_2_-injury models. The relative densities of NF-κB and phosphorylated STAT3 bands were normalized to actin in the same samples.

Based on the observed elevation of TNF-α and IL-6 with exosomal treatment, the downstream effect of NF-κB and STAT3 transcription factors activation in the G_1_ phase of the cell cycle was explored. These activations support the propagation of proliferative signals during liver regeneration [17]. The activation of these factors was investigated by immunoblotting of NF-κB (p65 and p50) and phospho-STAT3 (TYR705) after treatment of exosomes in both xenobiotic-induced hepatocyte-injury models. Without exosomes treatment, APAP- and H_2_O_2_-treated cells exhibited low basal levels of NF-κB and phospho-STAT3 activity (Figure 5, lane 2 and 5, respectively). With exosomal treatment, NF-κB (both p50 and p65) and phospho-STAT3 activities were restored to the normal expression levels in APAP-induced injury (Figure 5, lanes 3 and 4). Interestingly, higher levels of induction of the two proteins were more prominent in the H_2_O_2_-treated cells (Figure 3, lanes 6 and 7).

### Exosomes upregulated the expression of cell-proliferation markers (at G_1_ and S phase) after APAP- or H_2_O_2_-induced injury

**Figure 6:**
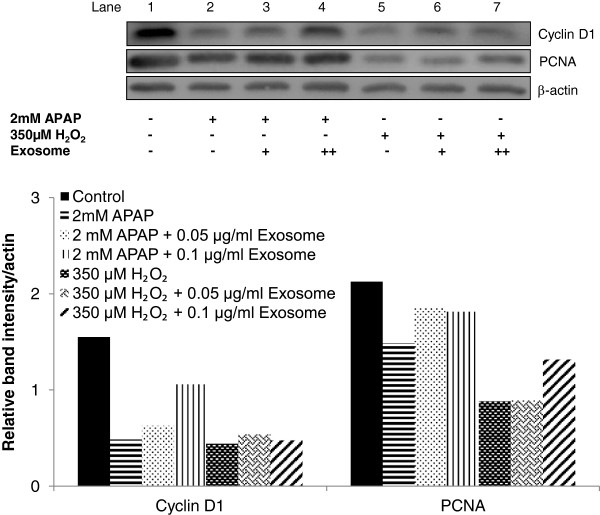
**Effect of exosomes in cell proliferation after APAP- or H**_**2**_**O**_**2**_**-induced injury in hepatocytes.** Expression of cyclin D1 and PCNA was determined by immunoblotting after 24-hour treatment in the APAP- or H_2_O_2_-injury model. The relative densities of cyclin D1 and PCNA bands were normalized to actin in the same samples.

After the observed restoration of NF-κB and STAT3 signaling with exosomal treatment, we pursued the search for biochemical evidence of active cell cycling by monitoring cyclin D1 and PCNA. Exosomes treatment increased the expression of cyclin D1 and PCNA in a dose-dependent manner during APAP and H_2_O_2_ injury (Figure 6). This finding is consistent with our earlier results, which demonstrated that MSC-derived exosomes promoted hepatocyte regeneration and proliferation during acute injury.

### Exosomes protected hepatocytes from apoptosis by decreasing caspase 3/7 level while upregulating antiapoptotic gene *Bcl-*_*xL*_

**Figure 7:**
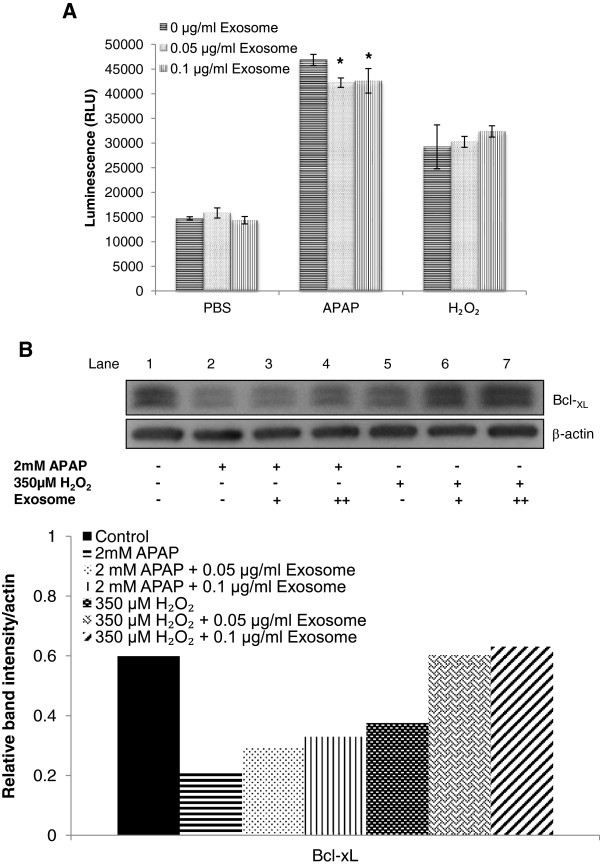
**Effect of exosomes on antiapoptosis in APAP- or H**_**2**_**O**_**2**_**-induced injury in hepatocytes. (A)** Caspase 3/7 was measured after 24-hour treatment of exosomes in APAP or H_2_O_2_ injury. **(B)** Expression of Bcl-_xL_ was determined by immunoblotting after 24-hour treatment in the APAP- or H_2_O_2_-injury model. *P < 0.05 versus APAP or H_2_O_2_ non-exosomes treatment group. The relative densities of Bcl-_xL_ bands were normalized to actin in the same samples.

Besides being a mitogenic transducer, STAT3 is also indicative of early response toward promotion of antiapoptotic activity. STAT3 could also suppress Fas-mediated liver injury either by a redox-dependent mechanism through expressing antiapoptotic genes such as Bcl-_xL_, Bcl-2 [18], or by a redox-independent mechanism through expressing Ref-1 [19] or anti-oxidative gene, manganese superoxide dismutase (MnSOD) [20]. To further determine if the anti-apoptotic effect was due to the upregulation of p-STAT3, caspase 3/7 activities were measured in this study. Both toxicant-only treatments resulted in significant increment of active caspase 3 and reduction of antiapoptotic gene, Bcl-_xL_, as compared with the control (Figure 7A,B). Similar to the favorable outcomes in cell viability, in APAP-injury model, exosomes treatment significantly suppressed the activity of caspase 3. This suppression was not observed in the H_2_O_2_-injury model. As for Bcl-_xL_, treatment of exosomes seemed to reverse the suppression observed in toxicant-only treatment. With higher exosomes concentrations, further increase in expression was demonstrated in both APAP- and H_2_O_2_- induced injury models (Figure 7B, lanes 4 and 7).

### Exosomes did not regulate the antiapoptotic effect through antioxidative genes

**Figure 8:**
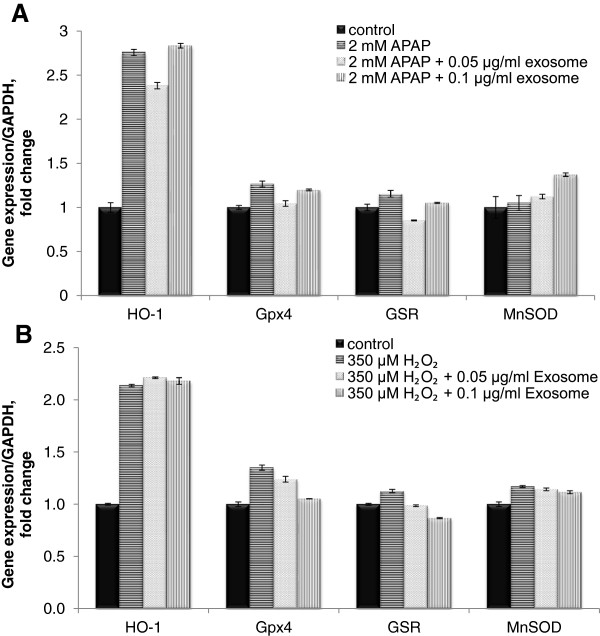
**Effect of exosomes on antioxidation in APAP- or H**_**2**_**O**_**2**_**-induced injury in hepatocytes.** Liver stress/defense-related genes expression in TAMH cells treated with or without exosomes concurrently in **(A)** 2 m*M* APAP or **(B)** 350 μ*M* H_2_O_2_ for 24 hours. All the expressions were determined by quantitative real-time PCR, normalized against GAPDH expression of the same sample and presented as fold-increase over the controls. Bars represent mean ± SEM.

Further to assess the antiapoptotic effect of exosomes mediated through the antioxidative pathway, mRNA expression of several stress/defense-related genes that are commonly involved in the oxidative pathway namely heme oxygenase-1 (HO-1), glutathione peroxidase 4 (Gpx4), glutathione reductase (GSR), and MnSOD were measured. In both injury models, only HO-1 mRNA expression was highly induced at the 24-hour time point of each toxicant treatment, whereas *Gpx4, GSR*, and *MnSOD* genes remained relatively unchanged, as compared with control. However, no significant difference appeared between the exosomes- and non-exosomes-treated groups (Figure 8A,B).

## Discussion

We hypothesized that the size-purified subset of exosomes derived from MSC-CM can play a role in mitigating xenobiotic-induced liver injury. As a proof of concept, we explored such effect by using classic models of liver toxicity (that is, CCl_4_-induced hepatic injury in mouse). We further considered the selectivity and the mechanism of the anticipated protection by using *in vitro* models of well-defined mechanisms of toxicity: APAP and H_2_O_2_. Accordingly, our experiments demonstrated that the MSC-derived exosomes accelerated *in vivo* liver regeneration in CCl_4_-injured mice. In addition, we found that exosomes consistently caused increased cell viability after the injury arising from both APAP- and H_2_O_2_.

In an *in vivo* study, CCl_4_ was used as a classic and well-established model of acute liver injury. The administration of MSC-derived exosomes was found to reverse CCl_4_-induced injury in mice with active proliferation of hepatocytes, most clearly indicated with the expression of PCNA, cyclin D1, and cyclin E. These proliferation effects were in line with the *in vitro* findings in which exosomes were able to sustain higher cell viability of hepatocytes after injuries cytotoxic doses of both APAP and H_2_O_2._ We qualified the regenerative potential of these exosomes by investigating the biochemical machinery behind the effect, beginning from the priming phase that triggers growth-arrested injured cells to reenter cell cycle.

IL-6 [21] and TNF-α [22] are prominent priming factors in initiating liver regeneration on injury by committing cells to cell cycling. Yamada *et al*. [22] reported defective DNA synthesis in TNFR-I-deficient mice after partial hepatectomy, whereas IL-6 injection restored the liver regeneration. Besides these, iNOS has also been demonstrated to mediate the formation of nitric oxide (NO) [23,24] and COX-2, which catalyzes the rate-limiting step in the synthesis of prostaglandins (PGs) that control the regenerative process. MIP-2 was also found to enhance hepatocytes proliferation through upregulating nuclear translocation of STAT-3 during the process of recovery from injury [25-27]. Our results demonstrated the increase in expressions of all these priming-phase factors after exosomes treatment alongside improved cell viability. Despite the use of a monoculture system comprising exclusively hepatocytes, we were able to pick up these cytokine perturbations, consistent with other similar liver cell-line studies reported in the literature [28,29].

TNF initiates liver regeneration by activating the NF-κB and IL-6-dependent pathway, which involves STAT3 transcription factor [30] in the G_1_ phase. In the current investigation, Western blot demonstrated that injured hepatocytes with exosomes treatment markedly increased the expression of NF-κB and STAT3 transcription factors. The IL-6/STAT3 pathway subsequently innervates cell-cycle progression from G_1_ to S phase, as shown in the upregulation of cyclin D1 and PCNA after exosomes treatment. In line with the priming factors and transcription factors, all these upregulated gene and protein expressions collectively support a restoration of overall cell viability, suggesting that the exosomes therapy likely mediates the liver repair from acute liver injury through induction of liver regeneration in the hepatocytes.

In the process of characterizing the mode of action of exosomes, we were aware of a possible differential response to the type of cellular injury invoked by the toxicants. Hence, two mechanistically distinct injury models were investigated. APAP is a well-studied hepatotoxicant that mediates the liver-injury pathway principally through generation of its reactive metabolite, *N*-acetyl-*p*-benzoquinoneimine (NAPQI) and subsequent binding to hepatic proteins [31], whereas H_2_O_2_ mediates liver injury solely through the oxidative-stress pathway. However, a common effect observed in both injuries is the manifestation of mitochondrial dysfunction, leading to apoptosis [32,33]. Because the exosomes demonstrated a protective or injury-repair effect in both models, albeit to a different extent, a possible effect in mitigating oxidative stress was investigated. Previously, STAT3 was also shown to regulate mitochondrial Bcl-_xL_ and MnSOD expression in inhibiting caspase 3 during apoptosis [18,20]. Our results demonstrated that the Bcl-_xL_ expression increased with higher expression of STAT3 after the treatment of exosomes, whereas caspase 3/7 expression was significantly decreased in the APAP-injury group treated with exosomes. This suggests that exosomes were able to alleviate some mitochondrial dysfunction as part of its plethora of protective mechanisms in the antiapoptosis pathway. However, exosomes did not demonstrate a role in regulating antioxidative genes, and hence did not mitigate injury via modulation of oxidative stress. These suggest that exosomes primarily assert antiapoptotic, proliferative, and regenerative hepatic responses to liver damage.

Follow-up work would be necessary to determine the hepatoprotective components within the exosomes. Exosomes have been shown to contain biologically active proteins, lipids, mRNA, and miRNA [34-36]. Lai *et al.* profiled a total of 857 proteins in the exosomes by using mass spectrometry and antibody array [37]. Here, few of the prominent proteins that are potential components involved in the liver-regeneration process were identified. Among them, IL6ST/gp130, TNFRSF1A/TNFR1, and CXCL2/MIP-2 proteins were found to be associated with the priming factors during liver regeneration. IL6ST plays an important role in mediating the IL-6/STAT3 pathway, which initiates the hepatocytes protection [38-40]. Likewise, CXCL2/MIP-2 enhances hepatocytes proliferation through upregulating STAT-3 during the process of recovery from injury [25-27]. These proteinaceous contents could be released from the exosomes intrahepatically, triggering the higher expressions of IL-6, TNF-α, and MIP-2 after treatment, as shown in our results.

Apart from the priming factors, HGF and hepatocyte growth factor receptor (HGFR) proteins, which are among the most potent stimulators of hepatocyte growth in liver regeneration [41,42], can also be found in the exosomes. These proteins might be working synergistically with the priming factors in accelerating and promoting liver regeneration, leading to a restoration of homeostasis. However, it remains to be determined whether miRNAs or other mRNAs found in the exosomes may have any impact on the proliferation effect of the hepatocytes.

## Conclusion

In conclusion, exosomes treatment demonstrated liver recovery after several toxicants-induced injuries. It is mediating this effect, likely through maintaining the liver homeostasis, that primarily includes inducing hepatocytes regeneration. This treatment may present a novel adjunctive therapy in drug-induced liver toxicity, in lieu of the limited availability of the conventional liver transplants. Its attractiveness also lies largely on its non-cell-based system, which reduces the risk of tissue incompatibility, while using an abundant and reproducible supply. This work supports further investigation to clarify the exact mechanism of action so that the intended therapeutic effect can be translated and optimized.

## Abbreviations

ALT: Alanine aminotransferase; APAP: acetaminophen; AST: aspartate aminotransferase; BSA: bovine serum albumin; CCl4: carbon tetrachloride; COX-2: cyclooxygenase-2; DMEM/F12: Dulbecco modified Eagle medium/Ham F12; DMSO: dimethylsulfoxide; FBS: fetal bovine serum; Gpx4: glutathione peroxidase 4; GSR: glutathione reductase; H&E: hematoxylin and eosin; H_2_O_2_: hydrogen peroxide; HGF: hepatocyte growth factor; HGFR: hepatocyte growth factor receptor; HO-1: heme oxygenase-1; HPLC: high-performance liquid chromatography; IL-6: interleukin 6; iNOS: inducible nitric oxide synthase; ip: intraperitoneal injection; is: intrasplenic injection; ITS: insulin, transferrin, selenium; MIP-2: macrophage inflammatory protein 2; MnSOD: manganese superoxide dismutase; MSC-CM: mesenchymal stem cell-conditioned medium; MTT: 3-(4,5-dimethylthiazol-2-yl)-2,5-diphenyltetrazolium bromide; NAPQI: N-acetyl-p-benzoquinoneimine; NO: nitric oxide; PBS: phosphate-buffered saline; PCNA: proliferating cell nuclear antigen; PG: prostaglandin; PMSF: phenylmethanesulfonyl fluoride; qRT-PCR: quantitative real-time polymerase chain reaction; TFF: tangential flow filtration; TGF-α: transforming growth factor alpha; TNF-α: tumor necrosis factor alpha.

## Competing interests

The authors declare that they have no competing interests.

## Authors’ contributions

CYT performed all the *in vivo* and *in vitro* assays including the biochemical and mechanism studies, data analysis, and interpretation, and drafted the manuscript. WW performed early characterization of exosomes in cell cultures. RCL and SKL contributed the MSC-derived exosomes and provided substantial contributions to the exosomes conception and information. YYD made substantial contributions to the design and interpretation of an *in vivo* model. HKH is the principal investigator for the overall project and supervised CYT in all aspects of this work. All authors read and approved the final manuscript.
